# Invasive Cyprinid Fish in Europe Originate from the Single Introduction of an Admixed Source Population Followed by a Complex Pattern of Spread

**DOI:** 10.1371/journal.pone.0018560

**Published:** 2011-06-03

**Authors:** Andrea Simon, Robert Britton, Rodolphe Gozlan, Cock van Oosterhout, Filip A. M. Volckaert, Bernd Hänfling

**Affiliations:** 1 Evolutionary Biology Group, Department of Biological Sciences, University of Hull, Hull, United Kingdom; 2 Centre for Conservation Ecology & Environmental Change, School of Conservation Sciences, Bournemouth University, Poole, Dorset, United Kingdom; 3 Central Laboratory of Animal Diversity and Systematics, Katholieke Universiteit Leuven, Leuven, Belgium; 4 School of Environmental Sciences, University of East Anglia, Norwich, United Kingdom; Biodiversity Insitute of Ontario - University of Guelph, Canada

## Abstract

The Asian cyprinid fish, the topmouth gudgeon (*Pseudorasbora parva*), was introduced into Europe in the 1960s. A highly invasive freshwater fish, it is currently found in at least 32 countries outside its native range. Here we analyse a 700 base pair fragment of the mitochondrial *cytochrome b* gene to examine different models of colonisation and spread within the invasive range, and to investigate the factors that may have contributed to their invasion success. Haplotype and nucleotide diversity of the introduced populations from continental Europe was higher than that of the native populations, although two recently introduced populations from the British Isles showed low levels of variability. Based on coalescent theory, all introduced and some native populations showed a relative excess of nucleotide diversity compared to haplotype diversity. This suggests that these populations are not in mutation-drift equilibrium, but rather that the relative inflated level of nucleotide diversity is consistent with recent admixture. This study elucidates the colonisation patterns of *P. parva* in Europe and provides an evolutionary framework of their invasion. It supports the hypothesis that their European colonisation was initiated by their introduction to a single location or small geographic area with subsequent complex pattern of spread including both long distance and stepping-stone dispersal. Furthermore, it was preceded by, or associated with, the admixture of genetically diverse source populations that may have augmented its invasive-potential.

## Introduction

Population genetic studies of invasive species have become an instrumental component in the study of biological invasions [1], [2], [3]. The application of neutral molecular markers can elucidate demographic processes during the invasion process and identify colonization pathways and source populations [4], [5]. Such information not only facilitates management and prevention of further invasions but also provides a framework for studies on adaptive evolution during the invasion process [6]. An issue which has recently received much attention but remains poorly understood is the role of genetic diversity in determining the outcome of introductions of non-native species. Introductions of non-native species are often based on the release of a low number of founding propagules containing only a fraction of the genetic variation of the source populations [7]. Such reduced genetic diversity theoretically limits a species' ability to establish invasive populations invoking a genetic paradox [8], [9], [10], [11], [12]. Although many successful invasive species show reduced genetic diversity, recent research suggests that the effects of such bottlenecks are often counteracted by admixture among genetically divergent source populations [3], [13]. For example, multiple introductions have resulted in high genetic diversity of invasive crustaceans [14], fish [3], [15], [16], lizards [17] and plants [18]. Nevertheless, it is currently unknown whether such admixture is merely a side-effect of the invasion process or is actually facilitating the establishment process. Additional population genetic case studies, in combination with studies on ecologically significant traits and genome wide associations are crucial in providing answers to this question.

One of the most compelling fish invasions in the world today is arguably the topmouth gudgeon *Pseudorasbora parva* (Temminck and Schlegel, 1846). This small cyprinid species originating from East Asia was accidentally introduced into Europe in the 1960s in several countries around the Black Sea as part of contingents of Chinese carps for aquaculture [19], [20]. Since then, they have proved highly invasive through a combination of combination of sociological, economical and ecological factors that enabled their rapid human-assisted and natural dispersal throughout the continent. On introduction into a new water body, colonisation is facilitated by their tolerance of degraded aquatic ecosystems and their reproductive traits of early sexual maturity, batch spawning, high reproductive effort and paternal nest guarding that provide a high degree of invasive vigour [20], [21], [22]. Their capacity for subsequently forming high density populations can then result in sharing of common food resources with native fishes resulting in overlaps in trophic niche [23], with additional concerns over egg predation, disease transmission and facultative parasitism [22].

Whilst this *P. parva* invasion has been traced from the initial point of introduction towards the northern and western parts of Europe, as well as the south towards Turkey and Iran [22], its exact demographic scenario is currently unclear. They are now found in at least 32 countries with contrasting climates (e.g. Algeria, Austria, Poland, Spain), have invaded habitats with a wide range of ecological conditions and their life history traits differ considerably among invasive populations [22]. Possible (non-mutually exclusive) explanations of such variability are: (1) the existence of considerable phenotypic plasticity in life history traits and tolerance to environmental conditions, (2) a rapid evolutionary response, or (3) multiple independent introductions from divergent source populations [19], [22], [24]. Molecular markers have previously been employed to study such questions in other freshwater fish invasions in Europe [25], [26] and North America [7]. For example, using mitochondrial DNA, Vidal *et al*. (2010) [26] showed that the mosquitofish (*Gambusia holbrooki*) was introduced into Europe multiple times from USA. Some *P. parva* populations have also been identified as healthy carriers of pathogens, such as *Anguillicola crassus* [27] and the rosette agent *Sphaerothecum destruens* [28], [29]. It is currently unknown whether other invasive populations or native populations show a similarly low susceptibility to the rosette agent.

Consequently, *P. parva* appear to be a model fish well suited to studying the evolution of ecologically significant traits, disease resistance and the role of genetic diversity in establishment success. Thus, we perform a population genetic analysis of *P. parva* across their native and introduced ranges in order to test different models of colonisation and to determine levels of genetic variation across the invasive range of the species (see Material and Methods for specific hypothesis). This will provide a first population genetic framework for further evolutionary studies on the species.

## Materials and Methods

### Sampling scheme and hypothesis testing

**Figure 1:**
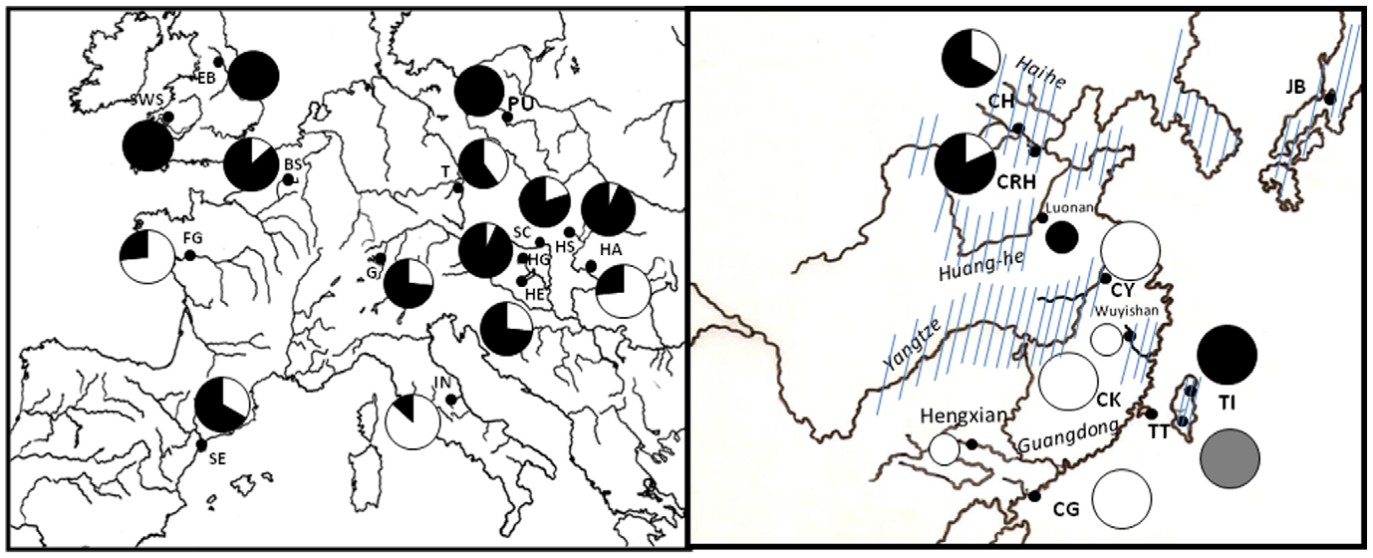
Distribution of *Pseudorasbora parva* samples sites in Europe (left) and in Asia (right), showing the species' native range. Pie charts represent the geographical distribution of major mtDNA lineages (see Figure 4). Lineage 1 = white, Lineage 2 = black, lineage 3 = grey. See Table 1 for population codes. Large pie charts represent samples collected in this study,small pie charts samples from Liu *et al.* 2010.

Samples were collected at a total of 22 sites, 14 in Europe and 8 in Asia (Table 1; Figure 1). Sample size was 15 for the majority of sites with the exception of three sites where 6–10 individuals where sampled. There was also a single sample (Japan) that comprised three individuals; it was excluded from all population-based analyses. The native range of the species is the East Asian sub-region, including the basins of the Huang He, Yangtze, Hai He and Amur Rivers, as well as some Japanese islands, Taiwan and the southern part of Korea [30], [31] and the sampling scheme covers most of the latitudinal space in this range, as well as spanning across the largest part of the European invasive range. The density of the coverage in the native range was appropriate to test some general demographic processes but not the identification of the exact location of potential source populations.

**Table 1:** Sample locations and sample sizes and geographical coordinates for native and invasive population.

Code	Population	N	Geographical co-ordinates	
CG	Guangdong, River, Zhuijang River basin, China	6	23°07′53″N	113°15′59″E
CH	Huairou Reservior, Hai He River basin, China	15	40°18′46″N	116°36′36″E
CK	Kinmen Island population, China	6	24°26′11″N	118°21′27″E
CRH	River Hai He, Hai He River basin, China	15	39°07′15″N	117°12′54″E
CY	Wuhan, Yangtze River Basin, China	10	29°58′20″N	113°53′29″E
JB	Lake Biwa, Yodo River basin, Japan	3	32°20′44″N	136°10′15″E
TI	I-lan county, I-lan River, Lanyang River Basin. Taiwan	15	24°45′00″N	121°45′00″E
TT	Dajia River, Taichung county, Dajia River basin, Taiwan	15	23°09′00″N	120°38′34″E
BS	Slangebeek nean Hasselt, Belgium	15	50°55′48″N	05°15′00″E
EB	Byland Abbey, Yorkshire, UK	15	54°12′10″N	01°09′35″W
FG	Grand Lieu, France	15	47°05′45″N	01°43′46″W
G	River Ammer, Wielenbach, Germany	15	47°52′11″N	11°09′00″E
HA	Hortobagy, Hungary	15	47°36′00″N	21°06′00″E
HE	Ederecsi-patak, Hungary	15	46° 48′04″ N	17° 23′16″E
HG	Gic, Hungary	15	47° 25′32″N	17°44′44″E
HS	Salyi-patak, Hungary	15	47°56′06″N	20°39′58″E
IN	Nestore, Italy	15	43°21′14″N	12°14′10″E
PU	Utrata River, Poland	15	50°35′50″N	18°09′32″E
SC	Vrakuna, Slovakia	15	47°49′24″N	18°49′16″E
SE	Ebro Basin, Spain	15	40°43′12″N	00°51′47″E
SWS	Sylen Lake, Llanelli, South Wales, UK	15	51°40′42″N	04°09′47″W
T	Blanice River, Vodnany, Czech Republic	15	49°08′52″N	14°10′32″E

Thus, the aim was to test three non-mutually exclusive models that were proposed to explain the spread of *P. parva* in Europe: i) ‘multiple source-sink’ model where several independent introduction events from genetically differentiated native source populations to separate European locations would have occurred without involving admixture; ii) ‘stepping-stone’ model [19] where introduction into a single geographical area would have been followed by gradual expansion from the original introduction; and iii) ‘long-distance’ model [22] where introduction into a geographical area would have been followed by long-distance translocation within Europe. Furthermore, it was tested whether iv) the invasive populations show signs of a genetic bottleneck or v) might have resulted from an admixture between divergent source populations.

Population genetic theory predicts that these demographic processes will result in different patterns of genetic population structure and therefore molecular approaches can be used to test the likelihood of alternative models. Therefore a number of phylogenetic and population genetic analyses were carried out in order to test the results against the theoretical expectations for the scenarios outlined above. Note that some of these tests assume that a relatively clear phylogeographic subdivision exists in the native range. Therefore the first step was to carry out a network analysis in order test this assumption. Genetic distances and *F*-statistics were used to quantify the degree of differentiation between populations and nucleotide diversity, and haplotype diversity at a standard sample size was used to estimate within population variability. These analyses were complemented by coalescent simulations and a Bayesian estimation of effective population size. The results were then compared with theoretical expectations from the various models and scenarios:

- ‘multiple-source-sink’ model: genetic differentiation among invasive populations is high and similar to that found in the native range;
- ‘stepping stone’ model: genetic differentiation in the invasive range is lower than that in the native range, and there is a significant pattern of isolation-by-distance;
- ‘long-distance’ model: genetic differentiation in the invasive range is lower than that in the native range, and there is no pattern of isolation-by-distance;
- ‘genetic bottleneck’ scenario: genetic diversity of invasive populations, in particular haplotype diversity, is lower than that of the source populations; and
- ‘genetic admixture’: genetic scenario: genetic variation expressed in nucleotide diversity is higher than that of the source population. Furthermore, recent admixture increases the nucleotide diversity above that expected under equilibrium conditions.

### Molecular procedures

The fish were collected and stored in 98% ethanol. Genomic DNA was extracted from the caudal fin tissue using the HotShot method [32]. An approximately 700 bp long section of the mtDNA genome, containing the partial *cytochrome b* gene was amplified applying standard PCR techniques using Verity Thermal Cycler. Primers L15267 and_H15891Ph, previously described by Briolay *et al*. (1998) [33], were used. Thermal cycle amplifications were performed in 15 µL reactions, containing 1.5 µL 160 mM NH_4_, 1.5 µL 100 mM dNTPs, 0.4 µL 50 mM MgCl_2_, 0.075 µL *Taq* polymerase, 0.3 µL each of primers L15267 and H15891Ph, 9.425 µL PCR water and 1.5 µL of template DNA. Cycle parameters were as follows: 2 min at 95°C; 45 s at 94°C, 45 s at 48°C, 1 min at 72°C; 10 min at 72°C. PCR products were directly sequenced in both directions using the PCR primers by Macrogen Inc. Forward and reverse sequences were aligned and edited using CodonCode Aligner [34], (GenBank accession numbers: JF489575-JF489887). Consensus sequences were imported into MEGA v. 4.1 [35] and aligned with ClustalW [36].

### Phylogenetic analyses and haplotype network

Phylogenetic relationships of haplotypes were reconstructed using the maximum composite likelihood method [37] in combination with Neighbour-Joining as implemented in MEGA v. 4.1 [35]. Furthermore we created a Maximum Likelihood tree, using the RaxML programme [38] using the GTR model optimised for each codon position. Branch support of both was obtained using non-parametric bootstrapping as percent of 1000 repeats and ML support values over 80% were added to tree nodes. Our aim using the phylogenetic tree approach was to show how distant haplotypes relate to major clades, rather than to provide definite resolution within clades.

In order to increase the geographic coverage, GenBank sequences from five *P. parva* individuals [39] sampled in the Minjiang River at Wuyishan (EU934500), the Pearl River at Hengxian (EU934501 and EU934502) and the Yellow River at Luonan (EU934503 and EU934504) were included in the phylogenetic analysis. Representatives of the main lineages of the cyprinid subfamily Gobioninae according to Tang *et al*. (2010) [40] were included as an outgroup using the same GenBank sequences as Tang *et al* (2010) [40].

A haplotype network was constructed using a median-joining algorithm in Network v. 4.5.10 [41]. Possible homoplastic sites (153, 195, 300, 462, and 585) were weighted down to 1 and all other nucleotide positions were weighted at 50 and we used an ε value of 0. Furthermore, transversions were weighted three times higher than transitions to decrease the likelihood of homoplastic substitutions [42]. A BLAST search of nucleotide sequences [43] was performed in order to confirm that all sequences belonged to *P. parva*.

### Population genetic data analysis

**Figure 2:**
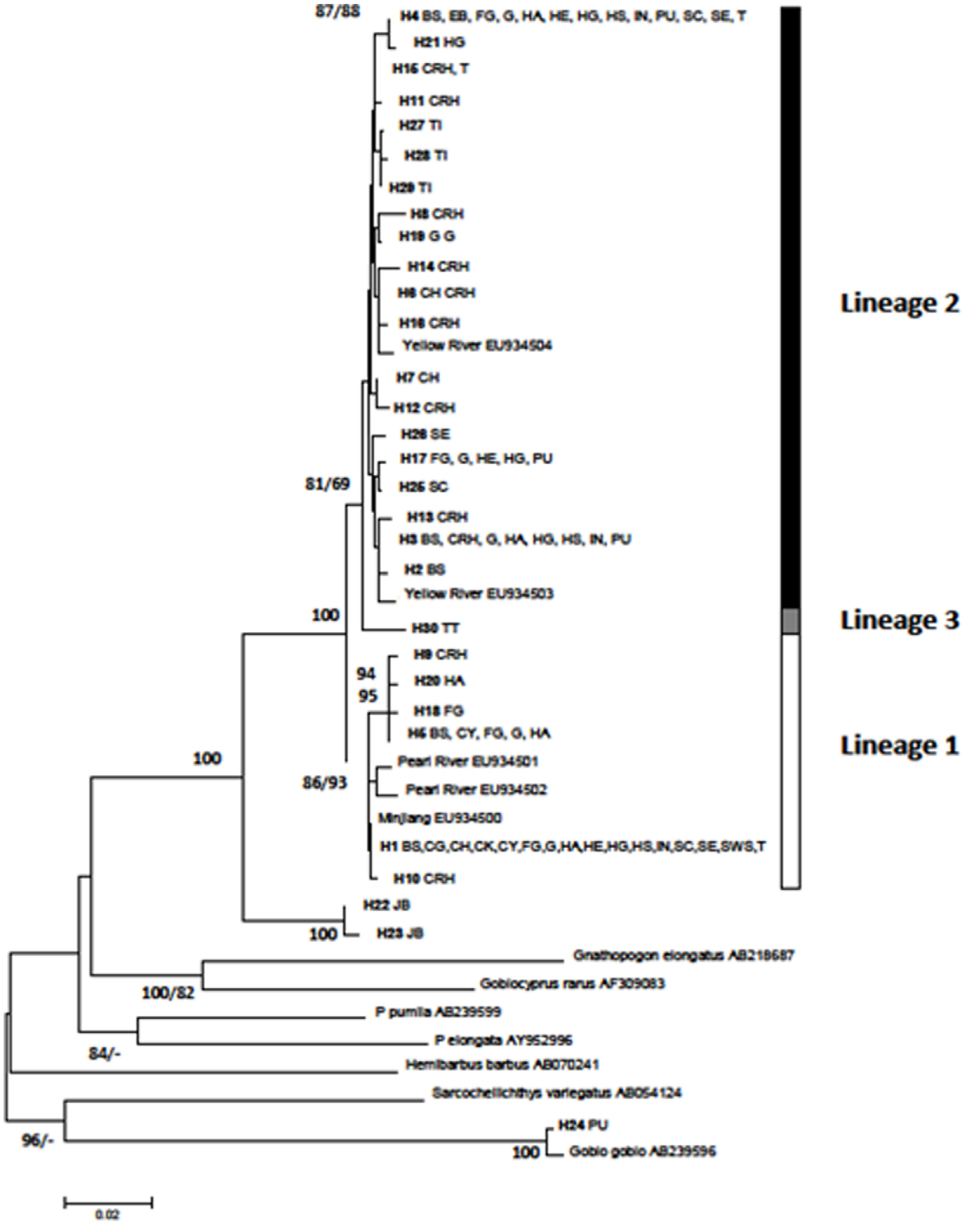
Estimates of effective population size (theta) of native populations based equilibrium assumptions.

DNaSP v. 4.5 [44] was used to estimate within population diversity (nucleotide diversity, π; haplotype diversity, Hs). Standardised measures of genetic diversity were calculated by resampling data sets 1000 times using a bootstrapping procedure [45], [46] based on the size of the smallest sample (6 individuals). Differences in genetic diversity between native and invasive populations were tested using a Mann-Whitney test. The invasive population PU was excluded from the comparison of π because it contained one highly divergent haplotype which is suspected to be derived from hybridisation with *Gobio gobio*. Coalescent based simulations as implemented in DNaSP were used to predict the expected relationship between haplotype diversity (*H*) and nucleotide diversity (*π*) under drift-mutation equilibrium and constant population size [47]. Effective population size of native populations assuming mutation-drift equilibrium and absence of migration among watersheds was estimated using MIGRATE-n v. 2.5 (Figure 2) [48]. The option Bayesian inference was used with the default search strategy settings. The rationale of this analysis was to estimate the populations size required to maintain the amount of genetic diversity found in the each population assuming mutation-drift-equilibrium.

Pairwise genetic differentiation among samples was computed as *F*_ST_ and *D*_XY_ (using Kimura two-parameter method, 1980 [49]) (Table 1, 2) using DNaSP v. 4.5 [44]. A multi-dimensional scaling (MDS) analysis based on *F*_ST_ was carried out in order to visualise the genetic relationship between samples. The average pairwise differentiation between native populations was compared to the average pairwise differentiation of invasive populations using a Mann-Whitney test. Isolation by distance (IBD) (Appendix, Table S3.) analysis was then used to test whether the ‘stepping-stone’ model could explain the spread of *P. parva* within Europe. Pairwise geographic distances among European sites were calculated as Euclidean distances. The theoretical expectation is that a significant correlation should only occur under the ‘stepping-stone’ model [50], [51]. Three different approaches were used. First, a ‘classical’ IBD analysis [52] was carried out to test the relationship between matrices of geographical distance and genetic differentiation (*F*_ST_) using a Mantel test (1000 permutations) as implemented in the software IBDWS v. 3.16 [53]. The genetic *F*_ST_ values were log-transformed to achieve a normal distribution. Second, a general linear model (GLM) was used to test the relationship between the geographic distance and genetic differentiation from the putative site of introduction. Third, a GLM was used to test the relationship the geographic distance from the putative site of introduction and genetic diversity of populations. Under a ‘stepping stone’ model, genetic diversity is expected to decrease with geographic distance to the original site of introduction, and hence, the genetic distance is expected to increase. The putative site of introduction was Nucet-Dombovita, Romania in the early 1960s [54], however around this time several other introductions took place into Hungary [22], so this population (HA) was used as reference population.

**Table 2:** Genetic diversity of *Pseudorasbora parva* populations.

Population	Group	*N*h	*H*	*H*_6_	π	code
CH	native	3	0.44	0.34	0.0056	green
CRH	native	11	0.96	0.75	0.0103	red
CK	native	1	0.00	0.00	0.0000	blue
CG	native	1	0.00	0.00	0.0002	purple
CY	native	2	0.46	0.35	0.0023	orange
TI	native	3	0.59	0.46	0.0011	brown
TT	native	1	0.00	0.00	0.0002	grey
BS	invasive	5	0.68	0.54	0.0067	white
EB	invasive	1	0.00	0.00	0.0000	white
FG	invasive	5	0.78	0.61	0.0109	white
G	invasive	6	0.84	0.66	0.0098	white
HA	invasive	5	0.62	0.48	0.0078	white
HE	invasive	3	0.59	0.46	0.0077	white
HG	invasive	5	0.62	0.49	0.0049	white
HS	invasive	3	0.25	0.20	0.0028	white
IN	invasive	3	0.34	0.27	0.0049	white
SC	invasive	3	0.67	0.53	0.0073	white
SE	invasive	3	0.67	0.52	0.0080	white
SWS	invasive	2	0.24	0.20	0.0012	white
T	invasive	3	0.68	0.55	0.0076	white
PU	invasive	4	0.60	0.47	0.0257	white

Columns represent populations, origin (native or invasive) number of haplotypes found in each population, observed haplotype diversity (H), mean haplotype diversity after bootstrapping based on sample size of 6 and nucleotide diversity (π) and colour code used in Figure 4.

### Approximate Bayesian Computation (DIY ABC)

Approximate Bayesian Computation (DIY ABC) [55] was used to estimate the relative likelihood of alternative scenarios of the initial introduction of the species into Europe. In the programme, reference tables (containing parameters based on known values) were used to compare the scenarios and the simulated datasets were then compared to the true values (Cornuet *et al*. 2008). DIY ABC is a computationally intensive approach and therefore only three simplified scenarios where chosen, which appeared most feasible after the initial population genetic analysis. An explicit rationale for choosing specific models will therefore be given in the Results section. The prior distribution of the coalescence time in the evolutionary scenario was partially informed by historical data, such as the date of the first introduction (Appendix, Table S3). The effective population size was set as uniform, 10 and 5×10^4^ individuals, covering the full range of biologically feasible values and the Kimura 2 parameters (1980) [49] mutation model was used. For each scenario 10^6^ datasets were simulated with the parameter values drawn from the prior distribution (Appendix, Table S3.). The relative likelihoods of the three scenarios were compared by using logistic regression on 1% of the closet simulated data sets.

## Results

### Phylogenetic and network analysis and distribution of haplotypes

**Figure 3:**
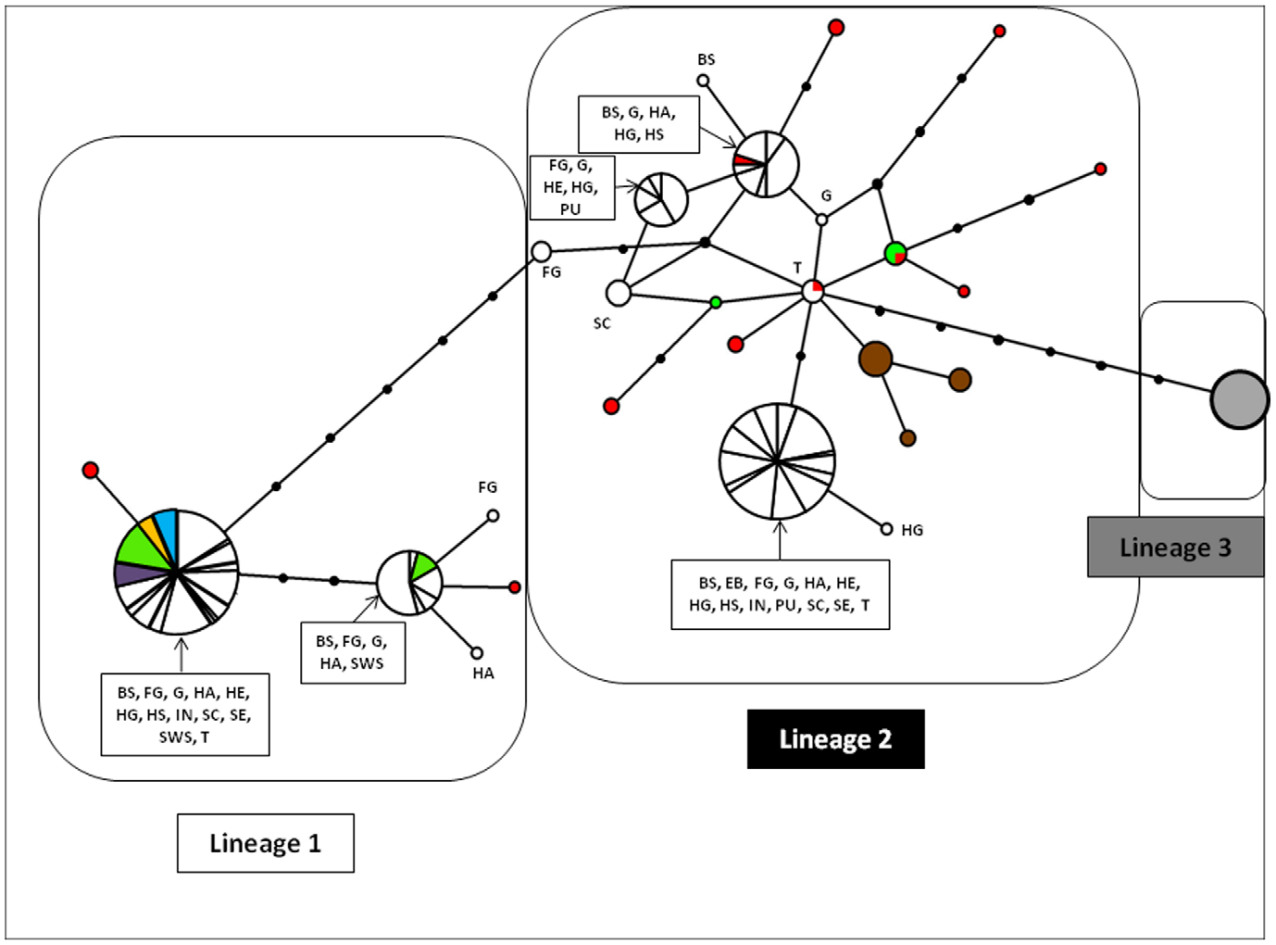
Phylogenetic relationship of haplotypes based on NJ analysis. First value on branches indicate ML support value, second value indicate non-parametric bootstrapping of the NJ-tree. Values are only given for support values >70%.

**Figure 4:**
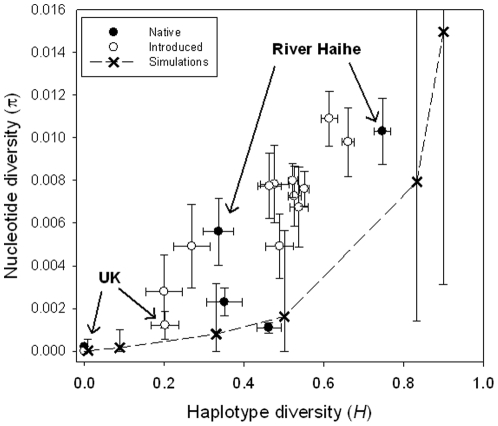
Medium joining network of *cyt*b haplotypes from native and introduced populations of *Pseudorasbora parva*, excluding H22, H23 & H24. Adjacent haplotypes are connected through a single point mutation. Each circle represents a single haplotype and its diameter is proportional to the number of individuals with that haplotype. The colour codes represent the locations in which the haplotype is found, filled cricles (•) represents unsampled haplotypes.

A total of 30 haplotypes were identified using 310 sequences from 8 native and 14 introduced populations (Table 1). The phylogenetic relationship among haplotypes is shown in Figure 3. Both NJ and ML methods yielded the same topology, hence only the NJ tree is displayed but ML support values were added to tree nodes. The two Japanese haplotypes, H23 and H24 were closely related to each other and the phylogenetic analysis (Figure 3) showed that they formed a highly divergent sister group to the remaining *P. parva* haplotypes (sequences divergence ∼5–6%). One highly divergent haplotype found in the invasive Polish populations clustered closely to a sequence of *G. gobio*. This haplotype and the Japanese haplotypes were therefore not included in the network analysis. Thirteen haplotypes were found in the invasive populations, five of which were found in more than one invasive population and will be subsequently referred to as common haplotypes. Three of the common haplotypes and two of the rare haplotypes were also found in at least one native population. Three main lineages of *P. parva* haplotypes can be recognised outside of Japan (Figures 3 and 4); a highly diverse central lineage (lineage 2) and two peripheral lineages (lineages 1 and 3) that are separated from the central lineage by 6 and 7 mutations, respectively. Lineage 3 consists of a single haplotype which is fixed in one of the native Taiwanese populations. One native population (TI) sampled in this study and the yellow river sample from Liu *et al* (2010) [39] are restricted to lineage 2 but do not share haplotypes with invasive populations. Three native populations (CG, CK, CY) sampled in this study and the Minjiang sample from Liu *et al* (2010) are restricted to lineage 1 and these populations also share a common haplotype with most introduced populations. Furthermore the Pearl River samples from Liu *et al* (2010) [39] fall into lineage 1 but do not share haplotypes with native populations. Two native populations (CRH, CH, Figure 1), however, contained haplotypes from both lineage 1 and 2 but share few haplotypes with the invasive populations. These two populations are from the Hai He River basin at the northern margin of the species distribution. The invasive populations are widely scattered across the network and most populations contain highly divergent haplotypes from both lineage 1 and 2. Among the native populations, the Taiwanese and Japanese (TI, TT, JB) populations do not share haplotypes with any native or invasive populations.

### Diversity within populations

**Figure 5:**
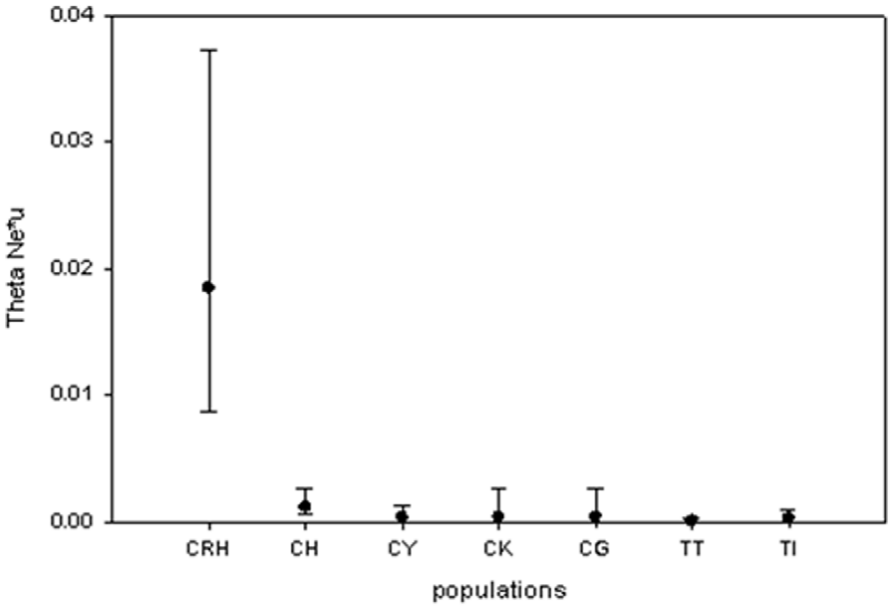
Plot of nucleotide (*π*) diversity versus haplotype (*H*) of the introduced (open circles) and native populations (solid circles). Also shown is the expected relationship between nucleotide diversity (±5–95% CI) and haplotype diversity of simulated populations (crosses) under mutation-drift equilibrium for populations. Excluded is the Polish population PU because its high value nucleotide diversity.

After bootstrapping to account for differences in sample size, the genetic diversity of the native populations varied widely among geographical regions. Whereas the two populations of the Hai He drainage showed relatively high diversity (*H* = 0.34, 0.76; *π* = 0.006, 0.010), the populations from other drainages of mainland China and Taiwan where much less variable (H = 0.00–0.46; π = 0.000–0.002). (Table 2, Figure 5). Genetic variation in introduced populations also varied considerably. The two recently established British populations showed low levels of variability (H = 0.00, 0.20; π = 0.000, 0.001) whereas the populations from continental Europe showed relatively high levels of variation (H = 0.20–0.66); π = 0.003–0.026). The highest nucleotide diversity was found in the Polish (PU) population (π = 0.026); this population contained one extremely divergent haplotype that clustered with a *G. gobio* haplotype, suggesting hybridisation and so was excluded from further comparisons. Overall genetic diversity in native populations (mean±SD; Hs = 0.27±0.29; π = 0.003±0.004) and invasive populations (mean±SD; Hs = 0.43±0.19; π = 0.008±0.006) was not significantly different (H, *P* = 0.108; π, *P* = 0.068). However, a more detailed analysis revealed that there were significant differences among certain groups of native and invasive populations. When the recently introduced UK populations were excluded from the analysis, both haplotype diversity and nucleotide diversity and were significantly higher in the invasive populations than native (*P* = 0.043 and, *P* = 0.014, respectively).

Next we simulated the nucleotide (π) and haplotype (H) diversity expected in a population that is in mutation-drift equilibrium with constant effective population size (*N*_e_), and we compared this to the empirical data (Figure 5). The simulations show that with increased *N*_e_, both H and π increase, which is predicted from theory, given that larger populations can harbour more nucleotide and haplotype diversity (Figure 5). However, the observed values of π for the introduced populations fall consistently above the theoretically predicted relationship between π and H. Thus, the introduced populations showed a relative excess of nucleotide diversity, given the observed haplotype diversity and assuming mutation-drift equilibrium. Similarly, some native populations also showed a relative excess in π compared to H (Figure 5). This pattern was inconsistent with a mutation-drift equilibrium and can be explained by admixture of populations with diverged nucleotide variation.

Maximum likelihood estimates of theta (*N*_e_ *μ*), using Migrate-n, differ by several orders of magnitude among native populations, ranging from 0.00006 for population TT to 0.01847 for population CRH (Figure 2). Using an average mutation rate for mtDNA of 1% per MY [56], this translates into effective population size estimates between approximately 10^3^ and 4.10^5^ individuals. This analysis is consistent with the previous simulation study as it shows that the standing nucleotide variation in some populations can only be explained by an exceedingly large effective population size, or more plausibly, by population admixture.

### Genetic differentiation and population structure

Pairwise genetic distance (*D*_XY_) ranged from 0 to 0.02715 and pairwise genetic differentiation (*F*_ST_) ranged from 0 to 1 (Appendix, Table S1), not including the Japanese (JB) population. The pairwise genetic distance among invasive populations (median *D*_XY_ = 0.009) was only marginally lower than that among native populations (median *D*_XY_ = 0.012), (*P*>0.3). Similarly, the native-invasive pairwise comparison expressed in *D*_XY_ (median *D*_XY_ = 0.012) (Appendix, Table S2) was neither significantly different from the genetic distance among native populations (*P*>0.3) nor from that among invasive populations (*P*>0.3).

In contrast, genetic differentiation (*F*_ST_) was considerably lower among the invasive populations (median *F*_ST_ = 0.21) than among the native populations (median *F*_ST_ = 0.58) (*P*<0.001). Furthermore, the *F*_ST_ between the native-invasive pairwise comparison (median *F*_ST_ = 0.53) was not significantly different from the genetic distance among native populations (*P* = 0.27), but it was significantly higher than that among invasive populations (*P*<0.001). This result is inconsistent with the ‘multiple-source-sink’ model, and supports both the ‘long-distance’ and ‘stepping stone’ models.

**Figure 6:**
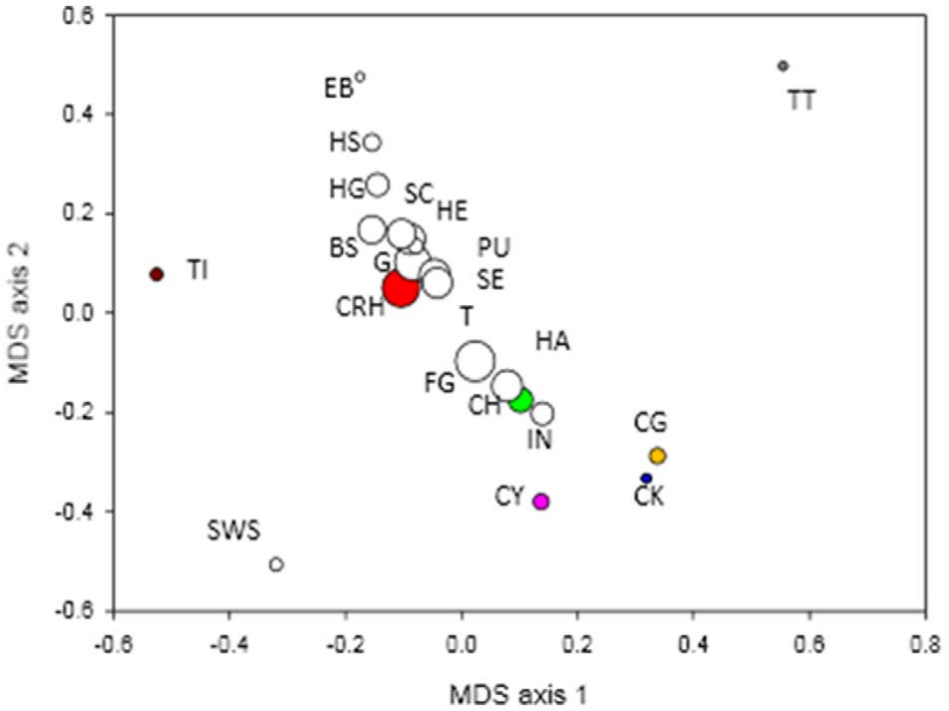
Plot of the first and second axis of a multidimensional scaling analysis based on pairwise *F*ST values among populations. Size of symbols is proportional to the nucleotide diversity of populations. Native populations are colour coded according to Table 1.

The multidimensional scaling analysis of the *F*_ST_ matrix (Figure 6) showed that most of the invasive populations cluster together with two native populations (CRH and CH). This cluster is surrounded by the remaining native populations and two introduced populations (EB and SWS). Overall, there appears to be a pattern that nucleotide diversity increases towards the centre of the plot, i.e. intermediate populations have the highest nucleotide diversity, which again indicates that these populations (invasives and the samples from the river Hai He) are genetically admixed.

A Mantel-test showed a significant relationship between genetic and geographic distance among the European populations (Z = −106685; *r* = 0.28, one sided *P*<0.05). However, when the recently introduced English (EB) and Welsh (SWS) populations were removed, there was no significant genetic isolation-by-distance relationship (Z = 9201, r = 0.06; one sided *P*>0.30). Regression analysis revealed no significant relationship between distance from source and genetic differentiation (*R* ^2^ = 0.005; P>0.03) or genetic diversity (*R* ^2^ = 0.018; P>0.03) respectively. This reveals that the English and Welsh populations are bottlenecked, resulting in the spurious isolation-by-distance signal obtained when including these samples in the Mantel test. However, across continental Europe, topmouth gudgeon does not show evidence of isolation-by-distance and so we conclude that the ‘long-distance’ model is most consistent with these data.

### DIY ABC

**Figure 7:**
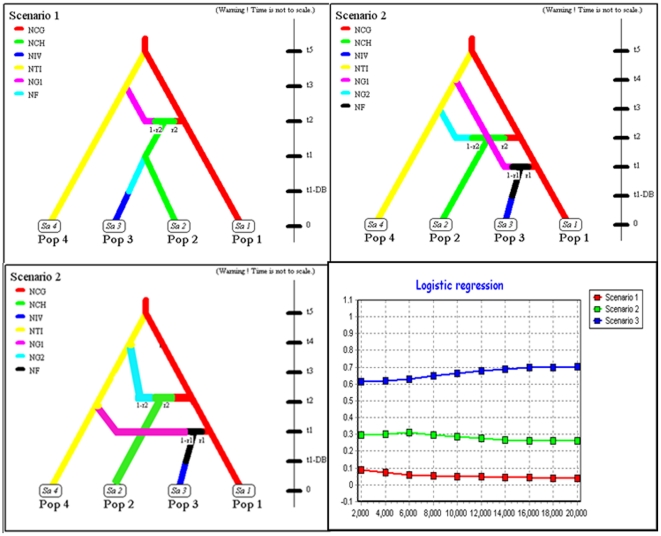
Graphic representation of the three competing invasion scenarios considered in the DIY ABC analysis. (Description of the scenarios are in the Results section.) Graph of linear regression, showing posterior probabilities of the scenarios.

Based on the geographic distribution of the haplotype lineages, samples were pooled into three native and one invasive population for which we considered three feasible evolutionary scenarios (Figure 7): (i) pop 1 (native populations of haplotype lineage 1; CG, CK, CY, Minjiang), (ii) pop 2 (admixed native populations from the river Hai He; CH, CRH), (iii) pop 3 (all invasive Hungarian populations; HA, HE, HG, HS), pop 4 (native populations of lineage 2; TI, Yellow River). The Hungarian populations were chosen to represent invasive populations because they were located in close proximity to the original site of introduction. In order to account for the unsampled variation in the native range in lineage 2, one or two ghost population (GH1, GH2) were included in the scenarios (represented as branches without terminal ends in Figure 7). All three scenarios assumed that a founder of size NF that lasted DB generations had event had taken place after introduction into Europe:

Scenario 1: The source of the invasive population (Pop 3) is the admixed Chinese population (Pop 2) which originates from an admixture of Pop 1 and a ghost population which split from Pop 4 at time t3.
Scenario 2: The invasive population (Pop 3) is a result of an admixture between pop 1 and an unsampled ghost population which split from pop 4 at time t4. Pop 2 evolved as in scenario 1. Scenario 3: same as Scenario 2 but the admixture of the pop 1 and GH2 populations took place before the admixture of Pop 1 and GH1.

A comparison of posterior probabilities of the three scenarios using local linear regression (Figure 7, Table 3) showed that scenario 1 showed the lowest support with probabilities lower than 0.1. The highest probability was shown for scenario 3. The posterior distribution of model parameters under the most likely scenario was used to make inferences about the timing of events during the colonisation process assuming a generation time of one year. The posterior density of the time of first introduction (*t*1) agrees with historical records (median = 47 generations, 95% credibility interval (CI) = 30–60). Full table of posterior distributions are given in the Appendix, Table S3.

**Table 3:** Output file of the Direct approach, relative proportion of each scenario found in the selected 500 closest dataset; Posterior probabilities of scenarios obtained through a logistic regression computed every 10% of the number of selected datasets.

Direct approachclosest						
		Scenario 1		Scenario 2		Scenario 3
50	0.24	[0.0000,0.6144]	0.3	[0.0000,0.7017]	0.46	[0.0231,0.8969]
100	0.27	[0.0000,0.6591]	0.28	[0.0000,0.6736]	0.45	[0.0139,0.8861]
150	0.2667	[0.0000,0.6543]	0.2533	[0.0000,0.6346]	0.48	[0.0421,0.9179]
200	0.26	[0.0000,0.6445]	0.26	[0.0000,0.6445]	0.48	[0.0421,0.9179]
250	0.24	[0.0000,0.6144]	0.268	[0.0000,0.6562]	0.492	[0.0538,0.9302]
300	0.2533	[0.0000,0.6346]	0.2533	[0.0000,0.6346]	0.4933	[0.0551,0.9316]
350	0.2514	[0.0000,0.6317]	0.2771	[0.0000,0.6695]	0.4714	[0.0339,0.9090]
400	0.2625	[0.0000,0.6482]	0.275	[0.0000,0.6664]	0.4625	[0.0255,0.8995]
450	0.2622	[0.0000,0.6478]	0.2733	[0.0000,0.6640]	0.4644	[0.0273,0.9016]
500	0.264	[0.0000,0.6504]	0.264	[0.0000,0.6504]	0.472	[0.0344,0.9096]

Logistic regressionclosest						
		Scenario 1		Scenario 2		Scenario 3
3000	0.0882	[0.0000,0.1868]	0.2861	[0.0806,0.4916]	0.6257	[0.3878,0.8636]
6000	0.058	[0.0138,0.1022]	0.314	[0.1675,0.4604]	0.6281	[0.4687,0.7874]
9000	0.0519	[0.0199,0.0840]	0.2917	[0.1797,0.4038]	0.6563	[0.5343,0.7784]
12000	0.0471	[0.0219,0.0724]	0.275	[0.1819,0.3680]	0.6779	[0.5767,0.7791]
15000	0.0431	[0.0224,0.0638]	0.2626	[0.1822,0.3430]	0.6943	[0.6070,0.7816]
18000	0.0398	[0.0224,0.0572]	0.2596	[0.1871,0.3322]	0.7005	[0.6222,0.7789]
21000	0.0373	[0.0222,0.0524]	0.2609	[0.1938,0.3280]	0.7018	[0.6296,0.7740]
24000	0.0363	[0.0226,0.0501]	0.2627	[0.1998,0.3255]	0.701	[0.6336,0.7684]
27000	0.0353	[0.0227,0.0478]	0.264	[0.2047,0.3234]	0.7007	[0.6372,0.7641]
30000	0.0341	[0.0226,0.0456]	0.2655	[0.2092,0.3219]	0.7003	[0.6402,0.7605]

## Discussion

The outputs of these analyses revealed that i) there are three evolutionary lineages of the topmouth gudgeon (*P. parva*) in the native range, two of which contributed to the colonisation of Europe; ii) most invasive populations have a higher genetic diversity than their native counterparts and a higher genetic diversity than expected under equilibrium conditions; iii) most native populations have a low genetic diversity typical for riverine fishes, an exception being samples from the Hai He river system which showed very high levels of genetic diversity, which under equilibrium conditions predict extremely high effective population sizes; and iv) the differentiation among invasive populations is much lower than among native populations.

### Population genetics of native populations

The existence of four highly divergent haplotype lineages indicates a long isolation among geographic populations of *P. parva*. An approximate estimation of divergence times using a standard molecular clock rate of 1% MY [56] suggests a separation of the Japanese from the Chinese and Taiwanese populations during the Miocene (5–6 MYA) which is consistent with [57]. Accordingly, the remaining lineages will have formed during early Pleistocene (1–1.5 MYA), which implies that multiple glacial refugia must have existed during the ice ages. Although the sampling scheme limits detailed phylogeograhic inferences, it is apparent that there is a clear geographic association of each lineage across most of the range, but also an area in Northern China where two lineages are found in sympatry. This becomes apparent when analysing genetic diversity within populations. Most *P. parva* populations from their native range showed low haplotype and nucleotide diversity and high levels of differentiation among river systems, which is consistent with the pattern found in many other small freshwater fishes of similar size, such as the European bullhead (*Cottus gobio* L.; [58], [59]) and guppies (*Poecilia reticulata* Peters; [60], [61]). The native populations from the northern range of the distribution were, however, characterised by extremely high genetic diversity, particularly the population CRH. Such high diversity is unusual among freshwater fish populations as they are usually highly structured and show low effective population sizes. Indeed, the effective population size was estimated as requiring approximately 400,000 individuals to maintain the levels of diversity observed in the CRH population and 24,000 individuals in population CH, based on a coalescence approach that assumes mutation-migration-drift equilibrium. Published estimates of effective population size in other freshwater fishes and our own estimates from the remaining native populations (*N*_e_<7000) are several orders of magnitude lower; for example, other cyprinid fishes range around 500 to 1000 individuals [62], guppies range from 100 to 900 [60] and European bullheads between 80 and 500 [63]. This suggests that the populations CRH and possibly CH are not at equilibrium but represent relatively recent secondary contact between divergent populations.

It is possible that the geographic area around the Hai He River basin represents a natural secondary contact zone between divergent phylogeographic lineages. Although the literature on this subject is relatively limited, it seems clear that high tectonic activity and sea level changes during the Pleistocene have created a complex phylogeographic pattern with little concordance among species [64]. Nevertheless, studies on other freshwater fish, such as *Hemibarbus lameo* [65] and *Salanx ariakensis* [66], found evidence that secondary contact between diverged populations from different major river systems took place during low sea levels at the end of the Pleistocene. Furthermore, the geographic area around the Hai He River basin represents a natural secondary contact zone between divergent phylogeographic lineages of the estuarine, flathead mullet (*Mugil cephalus*) [67], [68].

Alternatively, recent human translocations associated with aquaculture might have caused such an admixture; this may not be considered surprising given that freshwater aquaculture in this area of China is intense [69]. According to Gozlan *et al* (2010b) [22], a high volume of *P. parva* translocations have occurred in China prior to introduction in Europe. These cyprinid translocations coincided with the end of the Chinese civil war and the need for additional sources of animal proteins [22].

### Colonisation history

Our data showed that all invasive populations shared at least one of the four common haplotypes and that levels of genetic differentiation were low compared to native populations. Such a pattern would be expected if the invasive populations had spread from a single source. The alternative explanation of high levels of gene flow among initially differentiated invasive populations is extremely unlikely given that this would involve regular gene flow across watersheds. Therefore we reject the possibility that different European populations were independently colonised from divergent source populations (‘multiple-source-sink model’). However, a number of results indicated that the introduced populations represented an admixture of divergent source populations. First, the levels of nucleotide diversity of populations in continental Europe were, on average, higher when compared with native populations. Second, the nucleotide diversity of invasive populations was higher than expected from coalescent theory. Finally, the population structure analysis showed that the majority of the invasive populations and two (admixed) native populations occupied central positions in the MDS plot between divergent native populations. The main exceptions to this pattern were the two British populations, which showed a low genetic diversity and high levels of differentiation from other invasive populations, but were fixed for one or two common invasive haplotypes. These populations were founded relatively recently, most likely from sources in Germany [19], [22]. We suggest that this pattern is a result of secondary bottlenecks during spread and translocation within Europe. Our data do not enable us to distinguish whether the admixture event has happened before the introduction into Europe or shortly after the introduction, before the large scale expansion across Europe, but based on the assumption that a single introduction to the same geographical location is more parsimonious than two independent introductions we suggest that it is more likely that the admixture event has happened in the native range.

Although the sampling coverage in the native range was not comprehensive enough to pinpoint the exact location(s) which acted as a source of invasive European populations, some more general inferences can be drawn. The data outputs suggest that the invasive populations originate from mainland China rather than Taiwan or Japan. The haplotype distribution of invasive populations and populations from northern China raise the possibility that this area is the source of introduction. However, the DIY ABC analysis suggests that this is much less likely than a scenario where the invasive populations in Europe originate from an admixture between populations from lineage 1 (such as the Yangtze) and an unsampled population from lineage 2. Anecdotal reports suggest that *P. parva* were initially translocated to Romania and Hungary from the Yangtze River at Wuhan which is geographically close to our CY sample [22] and most likely originate from an aquaculture pond. Given our genetic results we suggest that these aquaculture populations consisted of a mixture of the local Yangtze population and fish wish were introduced from a different more northern river system possibly a tributary of the Yellow River.

The isolation-by-distance analysis indicated that both ‘stepping-stone’ and ‘long-distance’ processes might have contributed to the spread of *P. parva* in Europe. The weak but significant pattern of IBD across the whole data set was mainly caused by the highly bottlenecked British populations at the margin of the distribution. After excluding these two populations, none of the tests was significant. The ‘stepping-stone’ colonisation is therefore not likely to be the predominant process for the spread of the species in Europe. We suggest that long-distance dispersal must have played a major role, possibly as a consequence of fish transport associated with aquaculture. This is in agreement with Gozlan *et al.* (2010b) [22], who suggested a *P. parva* dispersal model showing dispersal distances of approximately 250 km from the 1970s to the end of the 1990s, followed by shorter dispersal of 20 km on average since 2000. Additional genetic analyses at the country level with greater resolution of the geographical pattern of haplotypes are likely to confirm this two-stepped invasion process.

### Evidence of hybridisation

A single individual from the Polish population contained a highly divergent haplotype. The phylogenetic analysis revealed that the sequence is very closely related to a published GenBank sequence of *G. gobio*. The genus *Gobio* belongs to the same cyprinid subfamily as Pseudorasbora, the Gobioninae and is a close European relative of *P. parva* [40]. Despite the close phylogenetic relationship, the two species show very different phenotypic appearances and misidentification is extremely likely given that only adults were sampled. Although laboratory experiments have not confirmed this, based on these results we therefore conclude that this indicates mitochondrial introgression and if analysis of nuclear data confirms this, we suggest that the invasive *P. parva* is able to hybridise with at least one native European species. This raises further concerns about the threat which *P. parva* poses to native European fish fauna and corroborates experimental evidence that hybrids between *P. parva* and another European cyprinid *Leucaspius delineatus* are possible [70].

### Conclusion

The European introduction of *P. parva* resulted from accidental releases from a human-induced faunal translocation [22]. Their European colonisation was initiated by the introduction to a single location or small geographic area it was preceded by, or associated with, the admixture of genetically diverse source populations. This adds to the existing evidence that many invasive populations show the genetic signature of admixture or of multiple introductions [3], [71]. Although the data available did not fully allow us to disentangle the source populations of the invasive populations, we now have a better perspective of the spread of the species within the native range and the introduction of the species into Europe. It remains to be tested how much of the observed phenotypic variation can be attributed to phenotypic plasticity, but the single origin model supported by our data makes it more likely that the disease resistance reported in some populations of *P. parva*, that potentially will lead to devastating consequences for native fishes [28], [29], is an ubiquitous feature of the invasive populations.

## Supporting Information

Table S1 Matrix of *F*_ST_ values of pairwise genetic comparisons between all populations.
(DOC)

Table S2 Matrix of *D*_XY_ values of pairwise genetic comparisons between all populations.
(DOC)

Table S3 Set of prior distributions based on historical and demographic data. Posterior probabilities of the selected scenarios in DIY ABC: mean, median and mode values and four quantiles of the posterior distribution. Prior and posterior values of mutation rate used in DIY ABC.
(DOC)
