# Supplementary material for: Invasive Cyprinid Fish in Europe Originate from the Single Introduction of an Admixed Source Population Followed by a Complex Pattern of Spread
Source: PLoS One. 2011 Jun 3;6(6):e18560. doi: 10.1371/journal.pone.0018560 (PMC3108587; doi:10.1371/journal.pone.0018560)
Supplement: Table S1 — Matrix of F ST values of pairwise genetic comparisons between all populations. (DOC) [file pone.0018560.s001.doc]

| **Appendix Table S1. Matrix of *F*_ST_ values of pairwise genetic comparisons between all populations**   |  | **BS** | **CG** | **CH** | **CK** | **CRH** | **CY** | **EB** | **FG** | **G** | **HA** | **HE** | **HG** | **HS** | **IN** | **PU** | **SC** | **SE** | **SWS** | **T** | **TI** | | --- | --- | --- | --- | --- | --- | --- | --- | --- | --- | --- | --- | --- | --- | --- | --- | --- | --- | --- | --- | --- | | **BS** |  |  |  |  |  |  |  |  |  |  |  |  |  |  |  |  |  |  |  |  | | **CG** | 0.74769 |  |  |  |  |  |  |  |  |  |  |  |  |  |  |  |  |  |  |  | | **CH** | 0.46104 | 0.20089 |  |  |  |  |  |  |  |  |  |  |  |  |  |  |  |  |  |  | | **CK** | 0.74769 | 0 | 0.20089 |  |  |  |  |  |  |  |  |  |  |  |  |  |  |  |  |  | | **CRH** | 0.08395 | 0.57273 | 0.25644 | 0.57273 |  |  |  |  |  |  |  |  |  |  |  |  |  |  |  |  | | **CY** | 0.69144 | 0.22222 | 0.17457 | 0.22222 | 0.51802 |  |  |  |  |  |  |  |  |  |  |  |  |  |  |  | | **EB** | 0.43448 | 1 | 0.76963 | 1 | 0.45977 | 0.92929 |  |  |  |  |  |  |  |  |  |  |  |  |  |  | | **FG** | 0.26745 | 0.29333 | 0.04509 | 0.29333 | 0.12943 | 0.20149 | 0.59542 |  |  |  |  |  |  |  |  |  |  |  |  |  | | **G** | -0.00956 | 0.58211 | 0.27341 | 0.58211 | 0.01664 | 0.52768 | 0.34244 | 0.10784 |  |  |  |  |  |  |  |  |  |  |  |  | | **HA** | 0.39093 | 0.16944 | -0.03407 | 0.16944 | 0.2346 | 0.12638 | 0.67532 | 0.01341 | 0.21045 |  |  |  |  |  |  |  |  |  |  |  | | **HE** | 0.08759 | 0.65064 | 0.3361 | 0.65064 | 0.11266 | 0.60012 | 0.23292 | 0.21001 | -0.0084 | 0.2542 |  |  |  |  |  |  |  |  |  |  | | **HG** | 0.06499 | 0.82478 | 0.5539 | 0.82478 | 0.19767 | 0.76729 | 0.16931 | 0.38197 | 0.06312 | 0.46982 | 0.01958 |  |  |  |  |  |  |  |  |  | | **HS** | 0.249 | 0.89909 | 0.6379 | 0.89909 | 0.31833 | 0.83359 | 0.02198 | 0.47257 | 0.18503 | 0.54341 | 0.06856 | 0.00539 |  |  |  |  |  |  |  |  | | **IN** | 0.5088 | 0.11358 | -0.02556 | 0.11358 | 0.33751 | 0.15137 | 0.8022 | 0.08955 | 0.32553 | -0.03727 | 0.37554 | 0.5942 | 0.67395 |  |  |  |  |  |  |  | | **PU** | 0.01281 | 0.48167 | 0.30202 | 0.48167 | 0.08471 | 0.46436 | 0.0553 | 0.20769 | 0.02221 | 0.25941 | 0.01701 | -0.01827 | 0.01574 | 0.32874 |  |  |  |  |  |  | | **SC** | 0.06726 | 0.69312 | 0.3789 | 0.69312 | 0.09204 | 0.6408 | 0.29483 | 0.22814 | -0.00938 | 0.31097 | -0.03159 | 0.02847 | 0.12263 | 0.43104 | 0.01387 |  |  |  |  |  | | **SE** | 0.14694 | 0.55026 | 0.21386 | 0.55026 | 0.11309 | 0.5034 | 0.39286 | 0.13614 | 0.02001 | 0.13979 | -0.01674 | 0.15028 | 0.2183 | 0.24952 | 0.07487 | 0.02743 |  |  |  |  | | **SWS** | 0.76717 | 0.85714 | 0.5267 | 0.85714 | 0.61589 | 0.44772 | 0.96798 | 0.34942 | 0.62916 | 0.42338 | 0.70816 | 0.83234 | 0.88918 | 0.56449 | 0.53079 | 0.73664 | 0.65 |  |  |  | | **T** | 0.17265 | 0.53714 | 0.17867 | 0.53714 | 0.09408 | 0.4891 | 0.42143 | 0.12594 | 0.02661 | 0.11522 | -0.01156 | 0.17662 | 0.24236 | 0.2244 | 0.0863 | 0.04427 | -0.04622 | 0.64662 |  |  | | **TI** | 0.43366 | 0.95763 | 0.68199 | 0.95763 | 0.31937 | 0.88213 | 0.90196 | 0.51416 | 0.35324 | 0.61997 | 0.43153 | 0.5227 | 0.68063 | 0.73977 | 0.18824 | 0.40772 | 0.45727 | 0.93267 | 0.44688 |  | | **TT** | 0.78701 | 1 | 0.84408 | 1 | 0.69872 | 0.94574 | 1 | 0.72821 | 0.71152 | 0.79592 | 0.76318 | 0.8398 | 0.90787 | 0.86922 | 0.48622 | 0.76984 | 0.7619 | 0.97456 | 0.76385 | 0.96032 | |  |  |  |  |  |  |  |  |  |  |  |  |  |  |  |  |  |  |  |  |
| --- | --- | --- | --- | --- | --- | --- | --- | --- | --- | --- | --- | --- | --- | --- | --- | --- | --- | --- | --- | --- | --- | --- | --- | --- | --- | --- | --- | --- | --- | --- | --- | --- | --- | --- | --- | --- | --- | --- | --- | --- | --- | --- | --- | --- | --- | --- | --- | --- | --- | --- | --- | --- | --- | --- | --- | --- | --- | --- | --- | --- | --- | --- | --- | --- | --- | --- | --- | --- | --- | --- | --- | --- | --- | --- | --- | --- | --- | --- | --- | --- | --- | --- | --- | --- | --- | --- | --- | --- | --- | --- | --- | --- | --- | --- | --- | --- | --- | --- | --- | --- | --- | --- | --- | --- | --- | --- | --- | --- | --- | --- | --- | --- | --- | --- | --- | --- | --- | --- | --- | --- | --- | --- | --- | --- | --- | --- | --- | --- | --- | --- | --- | --- | --- | --- | --- | --- | --- | --- | --- | --- | --- | --- | --- | --- | --- | --- | --- | --- | --- | --- | --- | --- | --- | --- | --- | --- | --- | --- | --- | --- | --- | --- | --- | --- | --- | --- | --- | --- | --- | --- | --- | --- | --- | --- | --- | --- | --- | --- | --- | --- | --- | --- | --- | --- | --- | --- | --- | --- | --- | --- | --- | --- | --- | --- | --- | --- | --- | --- | --- | --- | --- | --- | --- | --- | --- | --- | --- | --- | --- | --- | --- | --- | --- | --- | --- | --- | --- | --- | --- | --- | --- | --- | --- | --- | --- | --- | --- | --- | --- | --- | --- | --- | --- | --- | --- | --- | --- | --- | --- | --- | --- | --- | --- | --- | --- | --- | --- | --- | --- | --- | --- | --- | --- | --- | --- | --- | --- | --- | --- | --- | --- | --- | --- | --- | --- | --- | --- | --- | --- | --- | --- | --- | --- | --- | --- | --- | --- | --- | --- | --- | --- | --- | --- | --- | --- | --- | --- | --- | --- | --- | --- | --- | --- | --- | --- | --- | --- | --- | --- | --- | --- | --- | --- | --- | --- | --- | --- | --- | --- | --- | --- | --- | --- | --- | --- | --- | --- | --- | --- | --- | --- | --- | --- | --- | --- | --- | --- | --- | --- | --- | --- | --- | --- | --- | --- | --- | --- | --- | --- | --- | --- | --- | --- | --- | --- | --- | --- | --- | --- | --- | --- | --- | --- | --- | --- | --- | --- | --- | --- | --- | --- | --- | --- | --- | --- | --- | --- | --- | --- | --- | --- | --- | --- | --- | --- | --- | --- | --- | --- | --- | --- | --- | --- | --- | --- | --- | --- | --- | --- | --- | --- | --- | --- | --- | --- | --- | --- | --- | --- | --- | --- | --- | --- | --- | --- | --- | --- | --- | --- | --- | --- | --- | --- | --- | --- | --- | --- | --- | --- | --- | --- | --- | --- | --- | --- | --- | --- | --- | --- | --- | --- | --- | --- | --- | --- | --- | --- | --- | --- | --- | --- | --- | --- | --- | --- | --- | --- | --- | --- | --- | --- | --- | --- | --- | --- | --- | --- | --- | --- | --- | --- | --- | --- | --- | --- | --- | --- | --- | --- | --- | --- | --- | --- | --- | --- | --- | --- | --- | --- | --- | --- | --- |
|  |  |  |  |  |  |  |  |  |  |  |  |  |  |  |  |  |  |  |  |  |
|  |  |  |  |  |  |  |  |  |  |  |  |  |  |  |  |  |  |  |  |  |
