# Supplementary material for: Invasive Cyprinid Fish in Europe Originate from the Single Introduction of an Admixed Source Population Followed by a Complex Pattern of Spread
Source: PLoS One. 2011 Jun 3;6(6):e18560. doi: 10.1371/journal.pone.0018560 (PMC3108587; doi:10.1371/journal.pone.0018560)
Supplement: Table S2 — Matrix of D XY values of pairwise genetic comparisons between all populations. (DOC) [file pone.0018560.s002.doc]

| **Appendix Table S2. Matrix of *D*_XY_ values of pairwise genetic comparisons between all populations**   |  | **BS** | **CG** | **CH** | **CK** | **CRH** | **CY** | **EB** | **FG** | **G** | **HA** | **HE** | **HG** | **HS** | **IN** | **PU** | **SC** | **SE** | **SWS** | **T** | **TI** | | --- | --- | --- | --- | --- | --- | --- | --- | --- | --- | --- | --- | --- | --- | --- | --- | --- | --- | --- | --- | --- | | **BS** |  |  |  |  |  |  |  |  |  |  |  |  |  |  |  |  |  |  |  |  | | **CG** | 0.01336 |  |  |  |  |  |  |  |  |  |  |  |  |  |  |  |  |  |  |  | | **CH** | 0.01146 | 0.00351 |  |  |  |  |  |  |  |  |  |  |  |  |  |  |  |  |  |  | | **CK** | 0.01336 | 0 | 0.00351 |  |  |  |  |  |  |  |  |  |  |  |  |  |  |  |  |  | | **CRH** | 0.00931 | 0.01206 | 0.0107 | 0.01206 |  |  |  |  |  |  |  |  |  |  |  |  |  |  |  |  | | **CY** | 0.01466 | 0.00148 | 0.00479 | 0.00148 | 0.01308 |  |  |  |  |  |  |  |  |  |  |  |  |  |  |  | | **EB** | 0.00596 | 0.0148 | 0.01217 | 0.0148 | 0.00954 | 0.01628 |  |  |  |  |  |  |  |  |  |  |  |  |  |  | | **FG** | 0.01204 | 0.00771 | 0.00864 | 0.00771 | 0.01218 | 0.00826 | 0.01347 |  |  |  |  |  |  |  |  |  |  |  |  |  | | **G** | 0.0082 | 0.01173 | 0.01061 | 0.01173 | 0.01023 | 0.01282 | 0.00746 | 0.0116 |  |  |  |  |  |  |  |  |  |  |  |  | | **HA** | 0.01197 | 0.00471 | 0.0065 | 0.00471 | 0.01185 | 0.0058 | 0.01206 | 0.00949 | 0.01117 |  |  |  |  |  |  |  |  |  |  |  | | **HE** | 0.00794 | 0.01107 | 0.01005 | 0.01107 | 0.01017 | 0.01255 | 0.00504 | 0.01179 | 0.0087 | 0.01044 |  |  |  |  |  |  |  |  |  |  | | **HG** | 0.00624 | 0.01404 | 0.0118 | 0.01404 | 0.00949 | 0.01552 | 0.00296 | 0.01279 | 0.00786 | 0.01202 | 0.00645 |  |  |  |  |  |  |  |  |  | | **HS** | 0.00635 | 0.01382 | 0.01159 | 0.01382 | 0.00961 | 0.0153 | 0.00143 | 0.01297 | 0.00773 | 0.01163 | 0.00565 | 0.00387 |  |  |  |  |  |  |  |  | | **IN** | 0.01187 | 0.00278 | 0.00513 | 0.00278 | 0.01149 | 0.00426 | 0.01244 | 0.00869 | 0.01092 | 0.00615 | 0.01014 | 0.01212 | 0.01182 |  |  |  |  |  |  |  | | **PU** | 0.01643 | 0.02478 | 0.02242 | 0.02478 | 0.01966 | 0.02613 | 0.0136 | 0.02309 | 0.01815 | 0.02263 | 0.017 | 0.01503 | 0.01447 | 0.0228 |  |  |  |  |  |  | | **SC** | 0.00751 | 0.01184 | 0.01037 | 0.01184 | 0.00968 | 0.01332 | 0.00515 | 0.01177 | 0.00846 | 0.01096 | 0.00727 | 0.00627 | 0.00573 | 0.01071 | 0.01671 |  |  |  |  |  | | **SE** | 0.00863 | 0.00888 | 0.00865 | 0.00888 | 0.01031 | 0.01036 | 0.00658 | 0.01093 | 0.00908 | 0.0092 | 0.00773 | 0.0076 | 0.00689 | 0.0086 | 0.0182 | 0.00784 |  |  |  |  | | **SWS** | 0.01711 | 0.00428 | 0.00721 | 0.00428 | 0.01501 | 0.00319 | 0.01908 | 0.00931 | 0.01487 | 0.00785 | 0.01535 | 0.01831 | 0.01809 | 0.00705 | 0.02868 | 0.01612 | 0.01316 |  |  |  | | **T** | 0.00868 | 0.00822 | 0.00805 | 0.00822 | 0.00989 | 0.0097 | 0.00658 | 0.01059 | 0.00895 | 0.00873 | 0.00759 | 0.00761 | 0.00686 | 0.00808 | 0.01822 | 0.00779 | 0.00746 | 0.0125 |  |  | | **TI** | 0.00692 | 0.01294 | 0.01054 | 0.01294 | 0.00838 | 0.01442 | 0.00559 | 0.01234 | 0.00843 | 0.01175 | 0.00777 | 0.0063 | 0.00608 | 0.01156 | 0.0165 | 0.00706 | 0.00837 | 0.01721 | 0.00787 |  | | **TT** | 0.01583 | 0.01974 | 0.01798 | 0.01974 | 0.01711 | 0.02122 | 0.0148 | 0.02005 | 0.017 | 0.01919 | 0.01634 | 0.01535 | 0.01513 | 0.01881 | 0.025 | 0.01579 | 0.01678 | 0.02401 | 0.01612 | 0.01382 | |  |  |  |  |  |  |  |  |  |  |  |  |  |  |  |  |  |  |  |  |
| --- | --- | --- | --- | --- | --- | --- | --- | --- | --- | --- | --- | --- | --- | --- | --- | --- | --- | --- | --- | --- | --- | --- | --- | --- | --- | --- | --- | --- | --- | --- | --- | --- | --- | --- | --- | --- | --- | --- | --- | --- | --- | --- | --- | --- | --- | --- | --- | --- | --- | --- | --- | --- | --- | --- | --- | --- | --- | --- | --- | --- | --- | --- | --- | --- | --- | --- | --- | --- | --- | --- | --- | --- | --- | --- | --- | --- | --- | --- | --- | --- | --- | --- | --- | --- | --- | --- | --- | --- | --- | --- | --- | --- | --- | --- | --- | --- | --- | --- | --- | --- | --- | --- | --- | --- | --- | --- | --- | --- | --- | --- | --- | --- | --- | --- | --- | --- | --- | --- | --- | --- | --- | --- | --- | --- | --- | --- | --- | --- | --- | --- | --- | --- | --- | --- | --- | --- | --- | --- | --- | --- | --- | --- | --- | --- | --- | --- | --- | --- | --- | --- | --- | --- | --- | --- | --- | --- | --- | --- | --- | --- | --- | --- | --- | --- | --- | --- | --- | --- | --- | --- | --- | --- | --- | --- | --- | --- | --- | --- | --- | --- | --- | --- | --- | --- | --- | --- | --- | --- | --- | --- | --- | --- | --- | --- | --- | --- | --- | --- | --- | --- | --- | --- | --- | --- | --- | --- | --- | --- | --- | --- | --- | --- | --- | --- | --- | --- | --- | --- | --- | --- | --- | --- | --- | --- | --- | --- | --- | --- | --- | --- | --- | --- | --- | --- | --- | --- | --- | --- | --- | --- | --- | --- | --- | --- | --- | --- | --- | --- | --- | --- | --- | --- | --- | --- | --- | --- | --- | --- | --- | --- | --- | --- | --- | --- | --- | --- | --- | --- | --- | --- | --- | --- | --- | --- | --- | --- | --- | --- | --- | --- | --- | --- | --- | --- | --- | --- | --- | --- | --- | --- | --- | --- | --- | --- | --- | --- | --- | --- | --- | --- | --- | --- | --- | --- | --- | --- | --- | --- | --- | --- | --- | --- | --- | --- | --- | --- | --- | --- | --- | --- | --- | --- | --- | --- | --- | --- | --- | --- | --- | --- | --- | --- | --- | --- | --- | --- | --- | --- | --- | --- | --- | --- | --- | --- | --- | --- | --- | --- | --- | --- | --- | --- | --- | --- | --- | --- | --- | --- | --- | --- | --- | --- | --- | --- | --- | --- | --- | --- | --- | --- | --- | --- | --- | --- | --- | --- | --- | --- | --- | --- | --- | --- | --- | --- | --- | --- | --- | --- | --- | --- | --- | --- | --- | --- | --- | --- | --- | --- | --- | --- | --- | --- | --- | --- | --- | --- | --- | --- | --- | --- | --- | --- | --- | --- | --- | --- | --- | --- | --- | --- | --- | --- | --- | --- | --- | --- | --- | --- | --- | --- | --- | --- | --- | --- | --- | --- | --- | --- | --- | --- | --- | --- | --- | --- | --- | --- | --- | --- | --- | --- | --- | --- | --- | --- | --- | --- | --- | --- | --- | --- | --- | --- | --- | --- | --- | --- | --- | --- | --- | --- | --- | --- | --- | --- | --- | --- | --- | --- | --- | --- | --- | --- |
|  |  |  |  |  |  |  |  |  |  |  |  |  |  |  |  |  |  |  |  |  |
