# Supplementary material for: Invasive Cyprinid Fish in Europe Originate from the Single Introduction of an Admixed Source Population Followed by a Complex Pattern of Spread
Source: PLoS One. 2011 Jun 3;6(6):e18560. doi: 10.1371/journal.pone.0018560 (PMC3108587; doi:10.1371/journal.pone.0018560)
Supplement: Table S3 — Set of prior distributions based on historical and demographic data. Posterior probabilities of the selected scenarios in DIY ABC: mean, median and mode values and four quantiles of the posterior distribution. Prior and posterior values of mutation rate used in DIY ABC. (DOC) [file pone.0018560.s003.doc]

**Appendix Table S3. Set of prior distributions based on historical and demographic data.**

| **Prior** | | **Posterior** | | | | | | |
| --- | --- | --- | --- | --- | --- | --- | --- | --- |
| **Parameter** | **Distribution** | **mean** | **median** | **mode** | **q025** | **q050** | **q950** | **q975** |
| NCG | Uniform  [10 – 50,000] | 5.95E+03 | 3.25E+03 | 1.40E+03 | 5.22E+02 | 6.81E+02 | 2.14E+04 | 3.02E+04 |
| NCH | Uniform  [10 – 50,000] | 3.12E+04 | 3.28E+04 | 4.93E+04 | 6.77E+03 | 8.95E+03 | 4.86E+04 | 4.93E+04 |
| NIV | Uniform  [10 – 50,000] | 2.36E+04 | 2.27E+04 | 1.42E+03 | 1.33E+03 | 2.27E+03 | 4.71E+04 | 4.87E+04 |
| NTI | Uniform  [10 – 50,000] | 9.19E+03 | 5.89E+03 | 2.49E+03 | 9.82E+02 | 1.32E+03 | 3.01E+04 | 3.72E+04 |
| NG1 | Uniform  [10 – 50,000] | 1.98E+04 | 1.70E+04 | 4.94E+03 | 1.13E+03 | 1.85E+03 | 4.57E+04 | 4.78E+04 |
| t1 | Uniform  [30 – 60] | 4.62E+01 | 4.68E+01 | 6.01E+01 | 3.02E+01 | 3.14E+01 | 5.92E+01 | 6.00E+01 |
| DB | Uniform  [1 - 20] | 1.13E+01 | 1.15E+01 | 1.20E+01 | 1.38E+00 | 2.06E+00 | 1.97E+01 | 2.00E+01 |
| NF | Uniform  [1 – 1,000] | 3.88E+02 | 3.34E+02 | 1.75E+02 | 5.05E+01 | 7.24E+01 | 8.86E+02 | 9.43E+02 |
| t2 | Uniform  [60 – 200] | 1.41E+02 | 1.46E+02 | 1.93E+02 | 6.53E+01 | 7.06E+01 | 1.96E+02 | 1.98E+02 |
| r2 | Uniform  [10^-3^ – 9.99x10^-1^] | 2.77E-01 | 2.58E-01 | 2.26E-01 | 9.68E-02 | 1.17E-01 | 4.91E-01 | 5.78E-01 |
| t3 | Uniform  [200 – 1,200] | 7.69E+02 | 8.00E+02 | 1.09E+03 | 2.40E+02 | 2.77E+02 | 1.17E+03 | 1.18E+03 |
| t5 | Uniform  [5,000 – 10^6^] | 1.40E+04 | 1.25E+04 | 1.02E+04 | 6.81E+03 | 7.42E+03 | 2.50E+04 | 2.94E+04 |
| NG2 | Uniform  [10 – 10,000] | 8.04E+03 | 8.66E+03 | 9.94E+03 | 2.69E+03 | 3.94E+03 | 9.91E+03 | 9.96E+03 |
| r1 | Uniform  [10^-3^ – 9.99x10^-1^] | 3.57E-01 | 3.36E-01 | 2.86E-01 | 8.98E-02 | 1.23E-01 | 6.65E-01 | 7.45E-01 |
| t4 | Uniform  [1,200 – 5,000] | 3.25E+03 | 3.30E+03 | 3.56E+03 | 1.36E+03 | 1.50E+03 | 4.82E+03 | 4.91E+03 |
| Mean mutation rate (per site per generation) | Uniform  1.00E-08 | 6.84E-007 | 7.14E-007 | 9.67E-007 | 2.32E-007 | 2.85E-007 | 9.79E-007 | 9.90E-007 |

**Posterior probabilities of the selected scenarios in DIY ABC: mean, median and mode values and four quantiles of the posterior distribution**

**Prior and posterior values of mutation rate used in DIY ABC.**
